# Crosslinked polyarylene ether nitrile film as flexible dielectric materials with ultrahigh thermal stability

**DOI:** 10.1038/srep36434

**Published:** 2016-11-09

**Authors:** Ruiqi Yang, Renbo Wei, Kui Li, Lifen Tong, Kun Jia, Xiaobo Liu

**Affiliations:** 1 Research Branch of Advanced Functional Materials, School of Microelectronics and Solid-State Electronics, High Temperature Resistant Polymer and Composites Key Laboratory of Sichuan Province, University of Electronic Science and Technology of China, Chengdu, 610054, China

## Abstract

Dielectric film with ultrahigh thermal stability based on crosslinked polyarylene ether nitrile is prepared and characterized. The film is obtained by solution-casting of polyarylene ether nitrile terminated phthalonitrile (PEN-Ph) combined with post self-crosslinking at high temperature. The film shows a 5% decomposition temperature over 520 °C and a glass transition temperature (*T*_g_) around 386 °C. Stable dielectric constant and low dielectric loss are observed for this film in the frequency range of 100–200 kHz and in the temperature range of 25–300 °C. The temperature coefficient of dielectric constant is less than 0.001 °C^−1^ even at 400 °C. By cycling heating and cooling up to ten times or heating at 300 °C for 12 h, the film shows good reversibility and robustness of the dielectric properties. This crosslinked PEN film will be a potential candidate as high performance film capacitor electronic devices materials used at high temperature.

With the rapid development of electric industry, compact, portable, and light weight electronic devices have attracted considerable attention from the material research community^1^,^2^,^3^. Many works have been reported on optimizing the properties of the electronic materials for their applications in electronic devices^4^,^5^,^6^,^7^,^8^,^9^,^10^,^11^, especially for the applications at high temperature^12^,^13^. Inorganic ceramics have been widely used in these fields for their unique properties^14^,^15^,^16^. However, several intrinsic defects, including brittleness, weak dielectric strength, difficult to process and/or extremely high processing temperature, have encountered when using these inorganic ceramics^17^,^18^. In recently years, high performance polymers and polymer based composites as ideal alternative candidates for these applications have been intensively investigated owing to their fascinating properties^19^,^20^,^21^,^22^,^23^,^24^,^25^,^26^.

Film capacitors is one of the most widely applied unit in electric devices. The demands to dielectrics for capacitor use are that the stable dielectric properties, moderate mechanical properties and great reliability in changing environment. Up to now, biaxially oriented polypropylene film (BOPP), polyethylene terephthalate (PET) and Poly(vinylidene fluoride) (PVDF) are the most widely used organic dielectric materials for energy storage film capacitors^27^,^28^,^29^,^30^,^31^. However, BOPP, PET and PVDF based capacitor can only work at temperature lower than 150 °C due to their low glass transition temperatures (*T*_g_). As a result, to accommodate the BOPP or PVDF in hybrid or electric vehicles, aerospace space power system and high-temperature electronics, additional thermal manage system, which keeps stable temperature of the system for its regular work, is indispensable to transfer the heat to the outer space for the high temperature devices. Usually, a cooling system is employed to keep the temperature below 150 °C. This cooling system will lead to the auxiliary cooling system and extra weight for the device, which is unacceptable in practical application. Undoubtedly, new candidates that can work over a broad temperature and frequency range are urgently demanded. Li *et al.* report a thermal stable and low dielectric loss composite which can be used up to 300 °C^32^. However, the complexity of preparation process restricts the application in commercial production. Polyarylene ether nitrile (PEN), a high-performance polymer, has attracted considerable attention in recent years owing to its outstanding properties, including high thermal stability, radiation resistance and excellent mechanical properties^33^. The possible applications of PEN as dielectric materials has been widely explored^34^. However, the application of the PEN at high temperature, especially at temperatures higher than 250 °C, has not been reported due to its intrinsic glass transition temperature is lower than that temperature.

In this study, we report the preparation and characterization of novel crosslinked PEN film that can be used as dielectric materials with broad operating temperature, moderate mechanical properties and ultrahigh thermal stability up to 380 °C. The film is self-crosslinked by the phthalonitrile groups capped at the ends of linear PEN. The thermal properties and dielectric properties, especially dielectric properties at high temperatures are studied in detail.

## Results and Discussion

**Figure 1:**
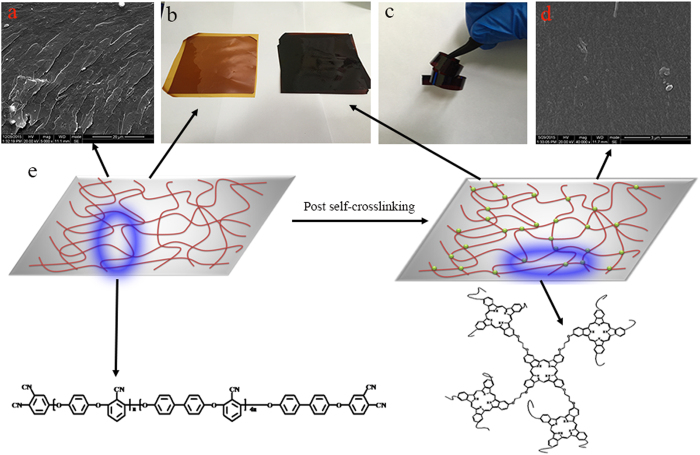
Morphology and cross-linking mechanism of the crosslinked PEN film. (**a**) morphology of the film before crosslinking, (**b**) the photos of the films before and after crosslinking, (**c**) the rolled up structure of the crosslinked PEN film, (**d**) morphology of film after crosslinking, (**e**) the preparation scheme of the crosslinked film and the structure of the PEN.

In this study, crosslinked polyarylene ether nitrile with ultrahigh stability up to 386 °C is fabricated through the scheme given in Fig. 1. The polyarylene ether nitrile terminated with phthalonitrile (PEN-Ph) is firstly synthesized in our laboratory according to our previous work^35^. Through simply curing with high temperature, the phthalonitriles capped at the ends of PEN self-crosslink and form the phthalocyanines as the crosslinking points in the system^36^,^37^. As the crosslinking points are at the ends of linear PEN, the obtained network is a PEN elastomer. The cross sectional micro-morphology of PEN-Ph is shown in Fig. 1a, relatively rough cross section is observed, indicating a thermoplastic material. After the post self-crosslinking, the film changes from brown to black (Fig. 1b). In addition, the film becomes rigid as the cross sectional micro-morphology of the crosslinked film is smooth and compact, as shown in Fig. 1d. Fortunately, the film is still flexible enough to be rolled up (Fig. 1c) and processed into different shapes, which is extremely important for its practical application.

**Figure 2:**
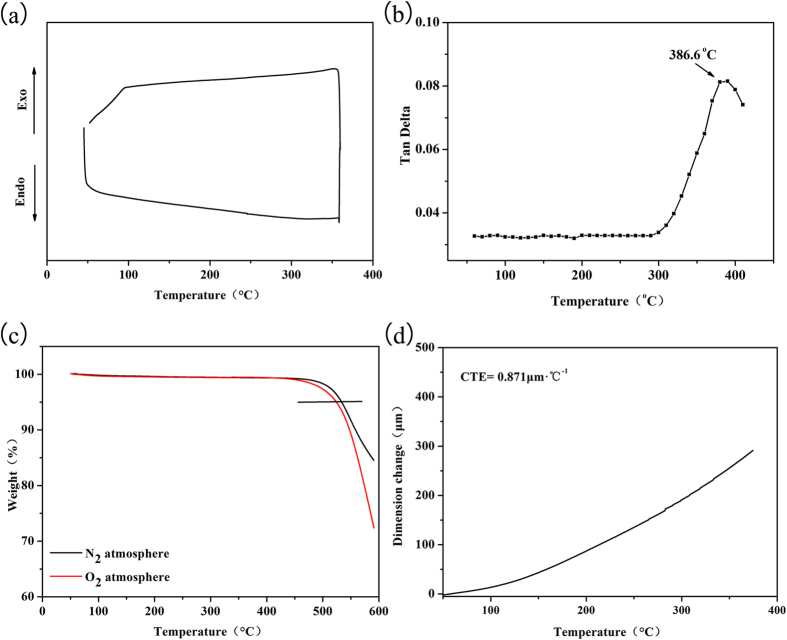
Thermal properties of the crosslinked PEN film (**a**) DSC curves during the heating and cooling scans, (**b**) tan delta of the crosslinked PEN film, (**c**) the TGA curves in oxygen and nitrogen atmosphere, (**d**) TMA curve of the crosslinked PEN film.

As a high-performance engineering polymer, PEN has been intensively studied in recent years owing to its outstanding properties including high thermal stability, good mechanical properties as well as radiation resistance. After crosslinking, these properties are further improved, which are needed in more strict circumstances. The thermal properties of the crosslinked PEN film are investigated by means of DSC, DMA, TGA as well as TMA. On the DSC curves, no obvious glass transition temperature (*T*_g_) is observed in the range of 50 to 360 °C (Fig. 2a) which is the detection limit of the instrument, indicating that the *T*_g_ of the film is higher than 360 °C. The DMA testing shows a peak for Tan delta at 386.6 °C (Fig. 2b), meaning the *T*_g_ of the crosslinked PEN film is around 386.6 °C. This super elevation of *T*_g_ is higher than that of most capacitor used organic dielectrics, such as BOPP and PET^38^,^39^. Figure 2c shows the TGA curves of the crosslinked PEN film. The crosslinked PEN film shows excellent thermal resistance with initial decomposition temperature (*T*_5%_) of 524.3 and 533.6 °C in N_2_ and O_2_ atmosphere, respectively. The unique dimension stability is studied by TMA, as shown in Fig. 2d. The coefficient of temperature expansion (CTE) is lower than 1 μm °C^−1^ over broad temperature range from 50 °C to 380 °C, while for BOPP, the lower melting temperature (164.6 °C) results in poor dimension stability^40^. These superior thermal properties of the crosslinked PEN film indicate that this novel material is more preferable for application at high temperature conditions.

**Figure 3:**
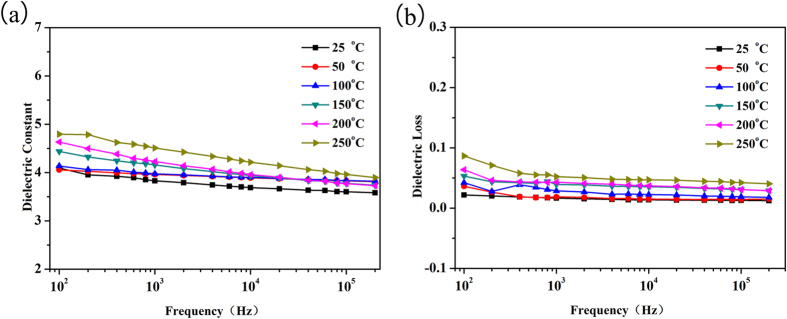
Dielectric properties of the crosslinked PEN film at vary conditions, (**a**) dielectric constant at different temperature, (**b**) dielectric loss at different temperature.

The dielectric property is one of the most important parameters of PEN which has been explored as dielectric and packaging materials in the microelectronics industry^41^. Usually, the dielectric constant of linear PEN decreases with the increasing of the measuring frequency due to the effect of the polarization relaxation^42^. While for the crosslinked PEN film, dielectric constant (~4.1) and dielectric loss (0.02) are almost same at room temperature with the increasing frequency (Fig. 3), fascinating properties for the practical application^43^. This result is mainly due to the fact that the movements of the macromolecular main-chains are restricted, and thus the orientation polarization is retained at all measured frequency^44^. When tested at higher temperature, the dielectric constant and dielectric loss increase a little (Fig. 3), and are only 4.3 and 0.05 at 1 kHz even at 250 °C. The increments of dielectric constant and dielectric loss are due to the higher activation energy of the system at higher temperature. When the temperature increases, the mobility of electrons is strengthened, leading to higher polarization of the system, and results in the increasing of dielectric constant. While as the macromolecular main-chains are still restricted in the network and the temperature is lower than the *T*_*g*_ of the system, this changing is negligible comparing with that of linear PEN^45^.

**Figure 4:**
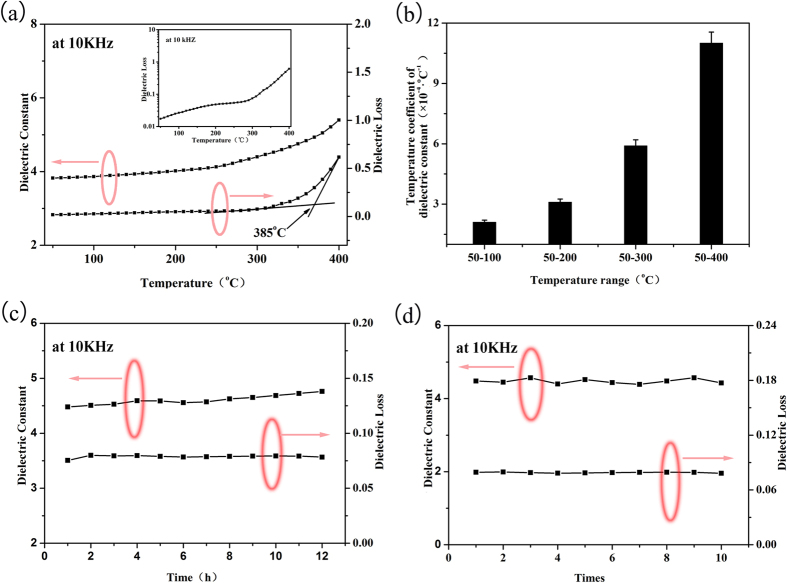
(**a**) The temperature dependencies of dielectric constant and dielectric loss of the crosslinked PEN film at 10 kHz; (**b**) The temperature coefficient of dielectric constant of the crosslinked PEN film at 10 kHz; (**c**) The dielectric properties of the crosslinked PEN film at 300 °C and at 10 kHz for different time; (**d**) The dielectric properties of the crosslinked PEN film at 300 °C and at 10 kHz on different cycling time.

The temperature dependencies of dielectric constant and dielectric loss of the crosslinked PEN film are further investigated. Tang *et al.*^46^,^47^ reported that the dielectric properties of a polymer are relatively stable before *T*_g_, and increase abruptly when the temperature is higher than the *T*_g_ of the polymer. When the temperature is below *T*_g_ of the polymer, the macromolecular motion is restricted, and is relatively weak. While the temperature is higher than *T*_g_ of the polymer, the macromolecular motion is enhanced and the polarization inside the system is strengthened, thus results in the increasing of the dielectric properties. As the film is a crosslinked network, the motion of the macromolecules is still restricted even the temperature is close to or higher than the *T*_g_ of it. As a result, the dielectric constant of the crosslinked PEN film at 10 kHz is stable before 300 °C, and increases slowly from 300–400 °C (Fig. 4a). Comparing with 20% increment of dielectric constant from 50 °C to 150 °C for BOPP, only 2.4% increment is observed for the crosslinked PEN film in the same temperature range^38^,^48^. While the competition between the motion of macromolecules and the restriction of the crosslinking still increases the dielectric loss of the system. As shown in the insert figure in Fig. 4a, when temperature is below 300 °C, the dielectric loss shows a slight increment from 0.017 to 0.064, which is favorable for the application in capacitor; while if the temperature further increases, the dielectric loss increases to 0.59 at 400 °C. Especially, an abruptly increment is observed on the curve of temperature dependencies of dielectric loss (Fig. 4a). According to the crossover point of tangent lines of the dielectric loss, the *T*_g_ of the crosslinked PEN film can be calculated to be 385 °C, consistent with the DSC and DMA results.

The temperature coefficient of dielectric constant, which can quantitative express the changing rate of dielectric constant with increasing of temperature, is determined by Equation (1):
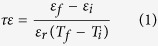
where *τ*_*ε*_ is the temperature coefficient of dielectric constant, *ε*_*f*_, *ε*_*i*_, and *ε*_r_ is the dielectric constant at finally temperature, initial temperature, and room temperature respectively, *T*_f_ and *T*_i_ is the finally temperature and initial temperature. According to Equation (1), the crosslinked PEN film shows excellent thermal stability as the *τ*_*ε*_of the film at 10 kHz is less than 0.001 °C^−1^ even at 400 °C, as shown in Fig. 4b. In addition, the current density at 10 kHz, the storage modulus, the breakdown strength and the energy density at different temperatures (in the supporting information) also confirm the thermal stability of this crosslinked PEN film. To make a clear view of the properties of the crosslinked PEN film, a comparison of the thermal and dielectric properties between BOPP and PEN-Ph self-crosslinked film is listed in Table S2.

For the practical application in electronic devices, excellent reversibility and robustness of the dielectric properties of the materials are necessary. The time dependence of the dielectric properties of the crosslinked PEN film is studied at 300 °C and at frequency of 10 kHz. As can be seen in Fig. 4c, both of the dielectric constant (~4.5) and dielectric loss (~0.07) of the crosslinked PEN film show excellent robustness even the film is consistently heated at 300 °C for up to 12 h. In addition, the dielectric properties of the crosslinked PEN film at 10 kHz are further investigated through repeat heating and cooling scans from room temperature to 400 °C. Concordant dielectric constant and dielectric loss are observed even by cycling up to ten times (Fig. 4d). The excellent thermal stability, reversibility and robustness of dielectric properties shown in Fig. 4 verify the long-term usage of this novel PEN based material at high temperature.

## Conclusion

Crosslinked polyarylene ether nitrile film as flexible dielectric material that can be used at temperatures as high as 380 °C was fabricated. By solution-casting of polyarylene ether nitrile terminated phthalonitrile (PEN-Ph) followed by post self-crosslinking at high temperature, this novel crosslinked PEN film can be prepared on a large scale. According to the results of DSC, DMA, TGA and TMA, the film showed superelevation of *T*_g_ and *T*_5%_, which are higher than 380 °C and 520 °C respectively. Due to the crosslinking, the film showed stable dielectric constant and low dielectric loss in the frequency range of 100–200 kHz and in the temperature range of 25–380 °C. The film showed excellent reversibility and robustness of the dielectric properties by cycling heating and cooling from room temperature to 400 °C up to ten times or heating at 300 °C for 12 h. Making use of these advantages, the crosslinked PEN film would be a promising candidate as the dielectric materials for high performance film capacitors electronic devices used at high temperature.

## Methods

### Materials

*N*-methyl-2-pyrrolidone (NMP) was purchased from Chengdu KeLong chemicals, Chengdu, China. Toluene and acetone were also purchased from KeLong chemicals, Chengdu, China. 4-Nitrophthalonitrile (99%) was purchased from Alpha chemicals (Dezhou) Co., Dezhou, China. Potassium carbonate (K_2_CO_3_), hydroquinone (HQ), biphenyl (BP), and 2, 6-dichlorobenzonitrile (DCBN) were commercially available products and used without further purification.

### Fabrication of crosslinked PEN film

The polyarylene ether nitrile terminated with phthalonitrile (PEN-Ph) was synthesized in our laboratory according to our previous work^20^, the detail of the synthesis of PEN-Ph can also be found in the supporting information. For the fabrication of crosslinked polymer films, PEN-Ph and certain amount of *N*-methyl-2-pyrrolidone (NMP) were added in a 100 mL three-necks round bottom flask charged with mechanical stirrer. The mixture was stirred and heated for 2.5 h to form a stable solution and then casted on a clean glass plate after cooled down to room temperature. The as-casted films were dried in an oven to remove the solvent. Furthermore, the dried films were transferred into a high temperature oven for post self-crosslinking at 280 °C, 300 °C, 320 °C, 340 °C and 360 °C every for 4 h, respectively. Finally, the crosslinked PEN films with thickness of 20–30 μm were obtained. The thermal properties and dielectric properties of the crosslinked PEN film were studied in detail. In addition, the other properties of the crosslinked PEN film, including the water absorption, mechanical properties as well as electrical performance were shown in the supporting information.

### Characterization

The cross-sectional morphologies of the crosslinked PEN films were observed with SEM (JEOL JSM-5900LV) operating at 20 kV. The thermal curing behavior of the crosslinked film was performed on TA Instrument DSC-Q100 with a heating and cooling rate of 10 °C/min from room temperature to 350 °C and in a nitrogen flow rate of 50 mL/min. Thermal gravimetric analysis of the crosslinked PEN film was obtained with a TA Instruments TGA-Q50 at a heating rate of 20 °C/min from room temperature to 600 °C under nitrogen and oxygen atmosphere. DMA test was carried out on TA-Q800 at a heating rate of 5 °C/min from 50 °C to 420 °C. TMA test was performed on a TA-Q400 and the dielectric properties were monitored according to the ASTM D150 on a HP4284A precision LCR meter. The mechanical properties were investigated by SANS CMT6104 Series Desktop Electromechanical Universal Testing Machine. Electric breakdown strength was tested by Dielectric Withstand Voltage Tester (ZJC-50KV). Electric displacements-electric field (D-E) loops were measured at 10 Hz with a Premier II ferroelectric test system (Radiant Technologies, Inc.) and the energy density of the materials in supporting was extracted from the D-E loops.

## Additional Information

**How to cite this article**: Yang, R. *et al.* Crosslinked polyarylene ether nitrile film as flexible dielectric materials with ultrahigh thermal stability. *Sci. Rep.* **6**, 36434; doi: 10.1038/srep36434 (2016).

**Publisher’s note**: Springer Nature remains neutral with regard to jurisdictional claims in published maps and institutional affiliations.

## Supplementary Material

Supplementary Information
