# Supplementary material for: Crosslinked polyarylene ether nitrile film as flexible dielectric materials with ultrahigh thermal stability
Source: Sci Rep. 2016 Nov 9;6:36434. doi: 10.1038/srep36434 (PMC5101497; doi:10.1038/srep36434)
Supplement: Supplementary Information [file srep36434-s1.doc]

Supporting Information

Crosslinked polyarylene ether nitrile film as flexible dielectric materials with ultrahigh thermal stability

Ruiqi Yang, Renbo Wei, Kui Li, Lifen Tong, Kun Jia* and Xiaobo Liu*

**Synthesis of PEN-Ph**: hydroquinone (HQ, 40.8 mmol), biphenyl (BP, 16.3 mmol) K_2_CO_3_ (0.24 mol) and 2, 6-dichlorobenzonitrile (DCBN, 0.20 mol) were added into a three-necks round bottom flask charged with mechanical stirrer. Then, *N*-methyl-2-pyrrolidone (NMP, 75 mL) and toluene (25 mL) were pour into the flask as solvent. After water-toluene was distilled off, the reaction mixture was heated to 150, 160, 170, and 180 ^o^C for 1 h, respectively. When the mixture was cooled down to 85 ^o^C, a certain amount of 4-nitrophthalonitrile (16.0 mmol) and K_2_CO_3_ (4 mmol) were added into the reaction mixture, then the temperature was keep at 85 ^o^C for 5 h. After that, the mixture was pour into dilute HCl solution to remove the K_2_CO_3_, and then the original product was purified by washing with alcohol, acetone and distilled water to yield the PEN-Ph.


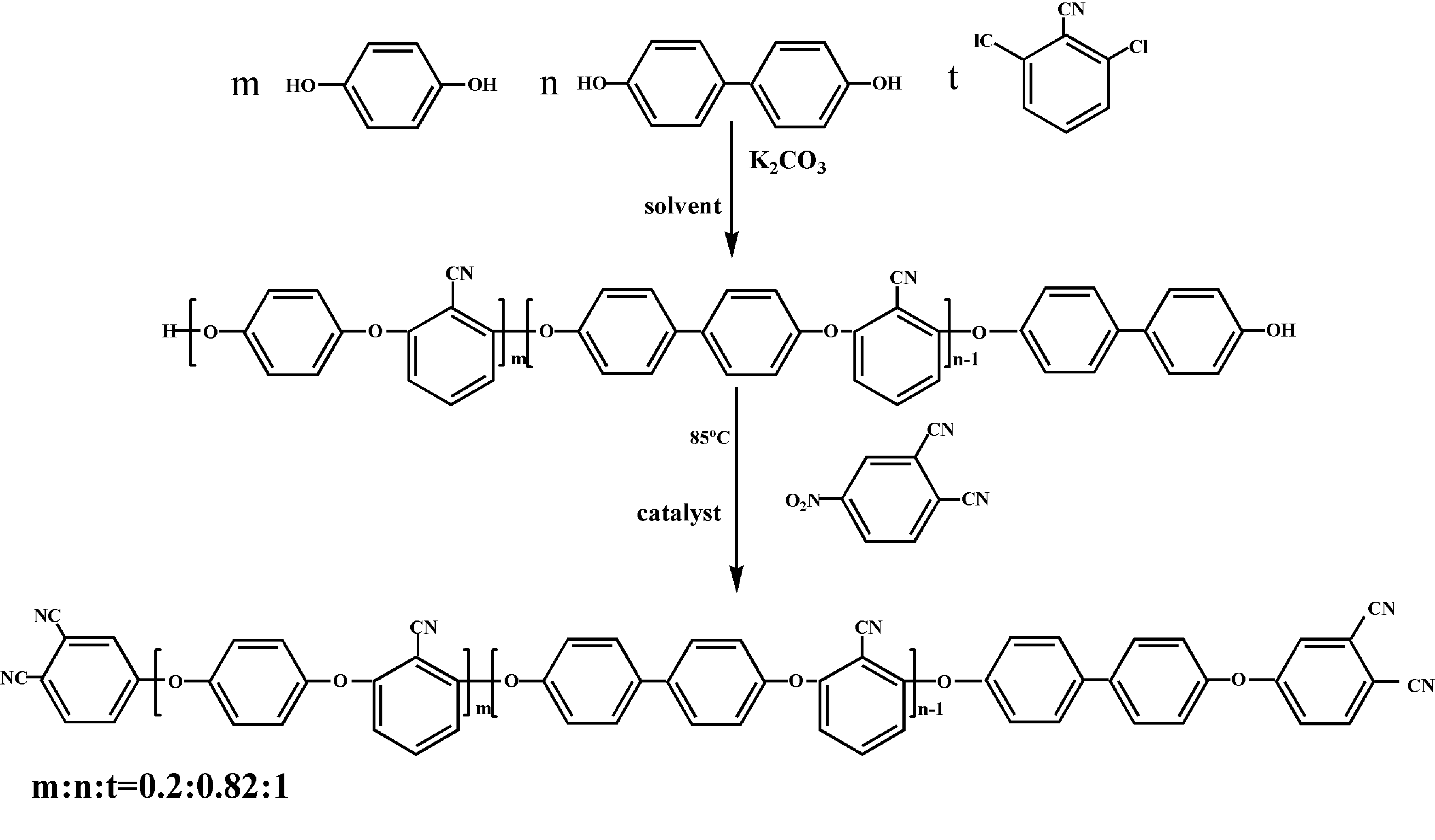


**Figure S1**. The schematic of the synthesis of PEN-Ph.

**Table S1.**The rate of water absorpation of the crosslinked PEN film at 25 ^o^C and 50 ^o^C (the rate of water absorpation of PI are 3.2% at 25 ^o^C, 4.6% at 50 ^o^C and 8.9% at 100 ^o^C).

| Temperature (^o^C) | 25 ^o^C | 50 ^o^C | 100 ^o^C |
| --- | --- | --- | --- |
| Water absorpation(%) | 0.93 | 1.40 | 3.52 |

The rate of water absorption is defined as:

$$\omega\%=\frac{w_{1}-w_{2}}{w_{2}}\times100\%$$

Where *w*_1_ is the weight of the samples after immersed in water for 24 h and *w*_2_ is the initial mass.


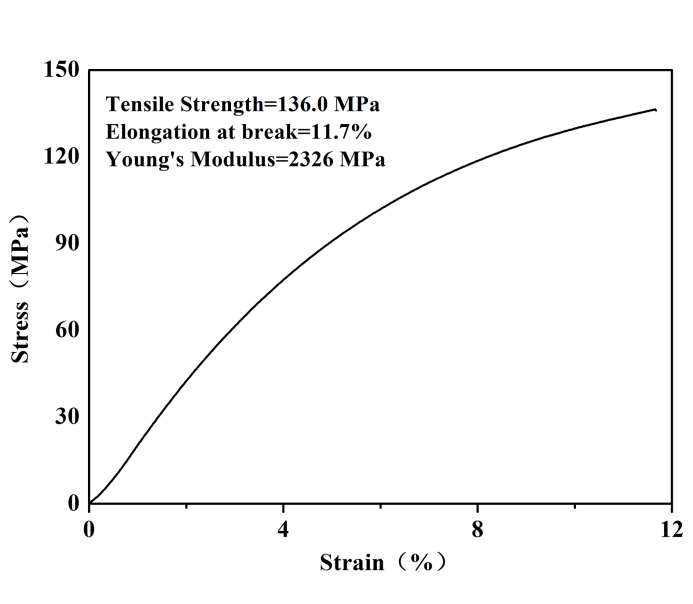


**Figure S2.**The stress-strain curves of the crosslinked PEN film at room temperature.


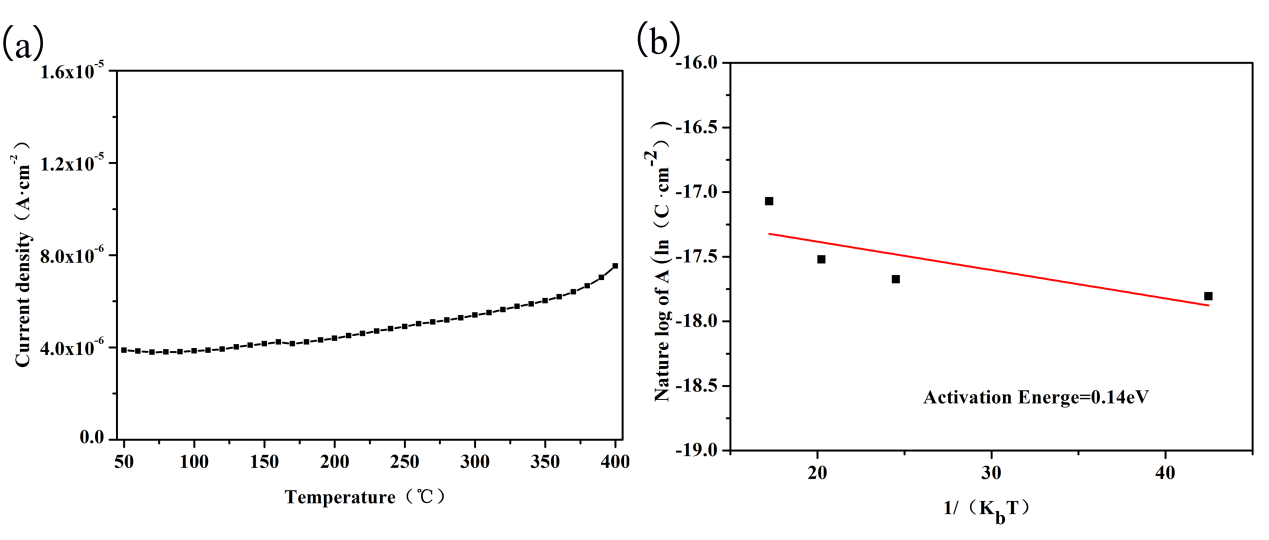


**Figure S3.** Electrical performance of the crosslinked PEN film. (a):The current density at 10 kHz as a founction of temperature; (b):Arrhenius plots of nature log of A versus 1/K_B_T.


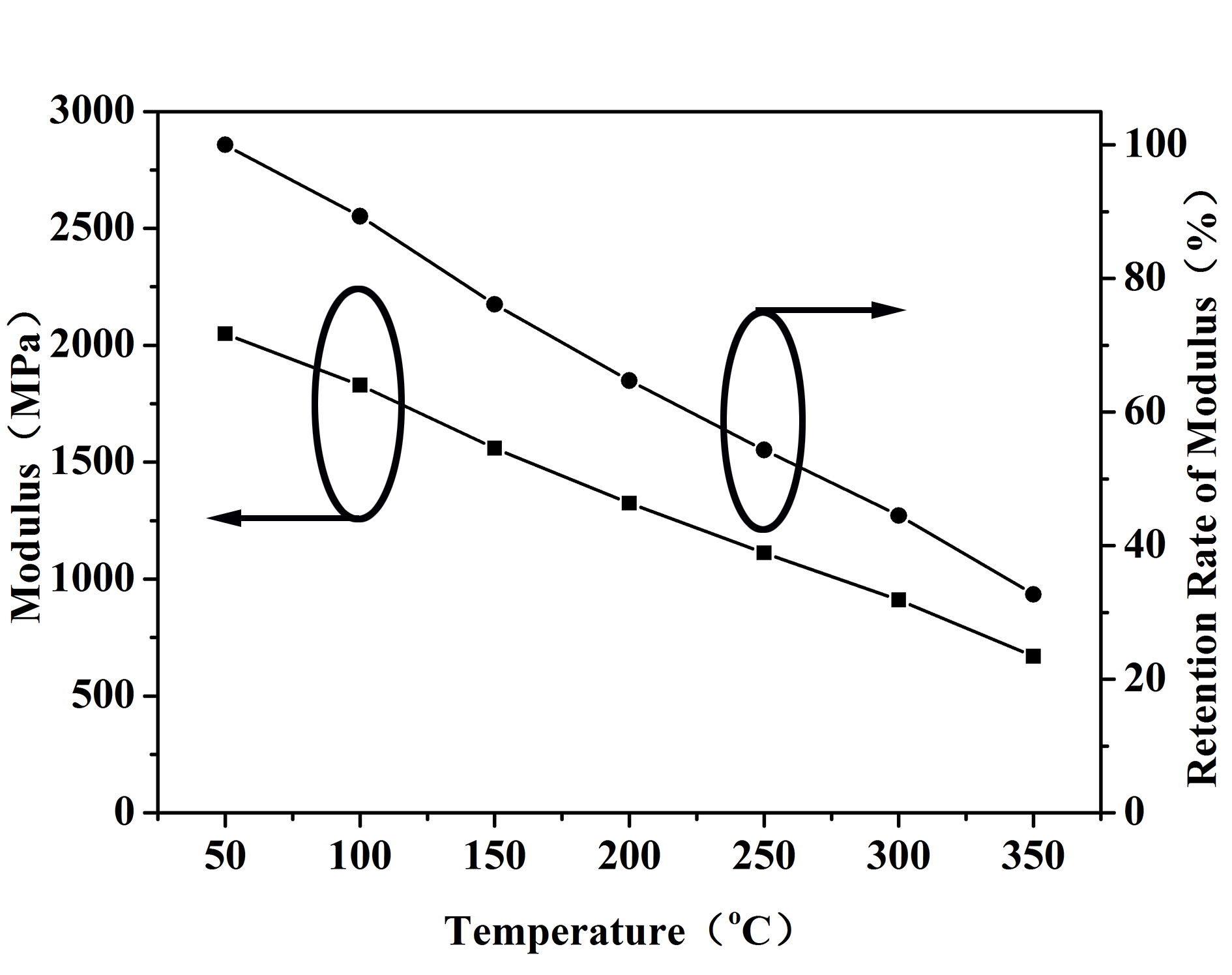


**Figure S4.** The storage modulus and retenion rate of modulus as a founction of temperature.


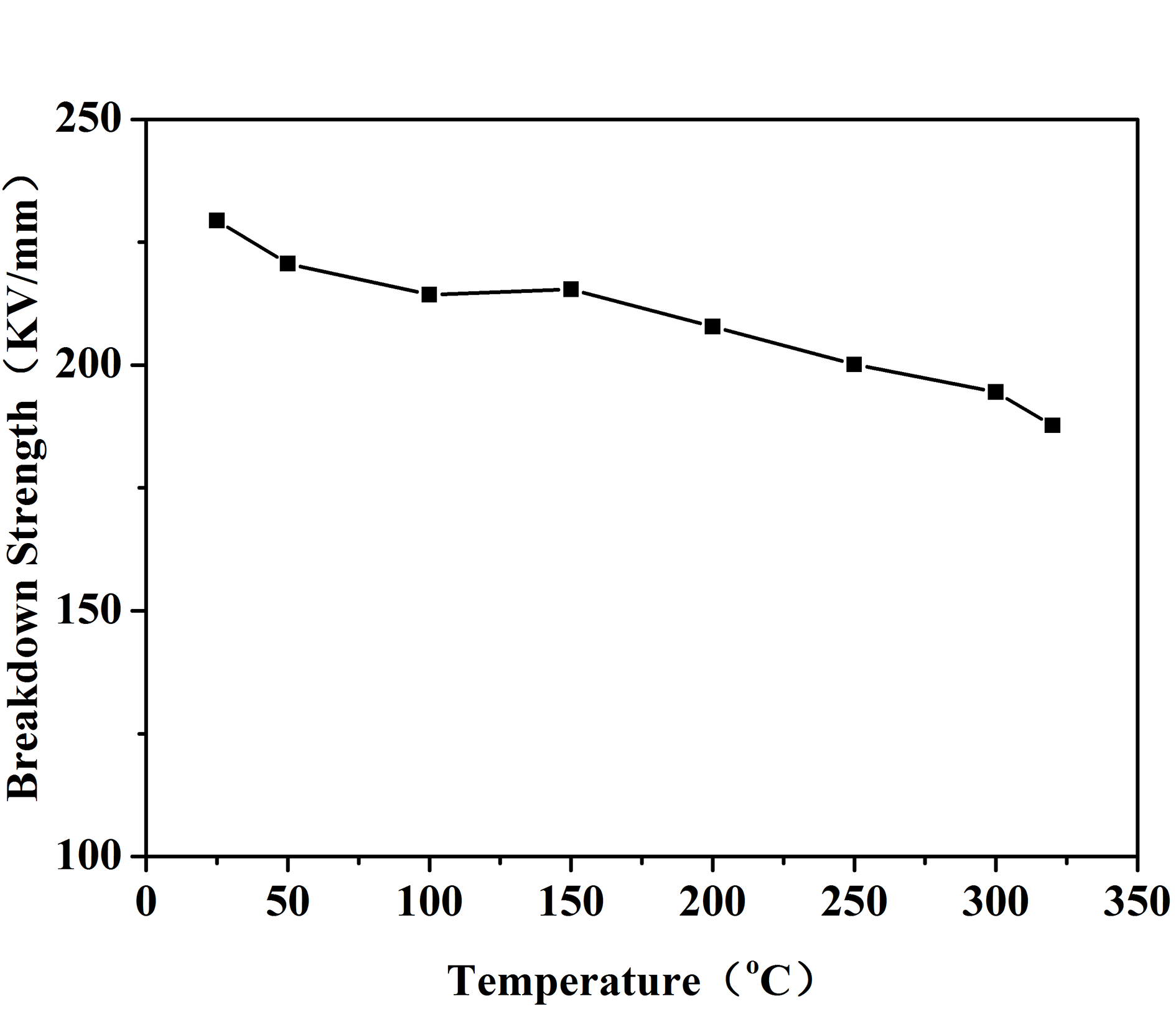


**Figure S5.** The breakdown strength as a function of temperature.


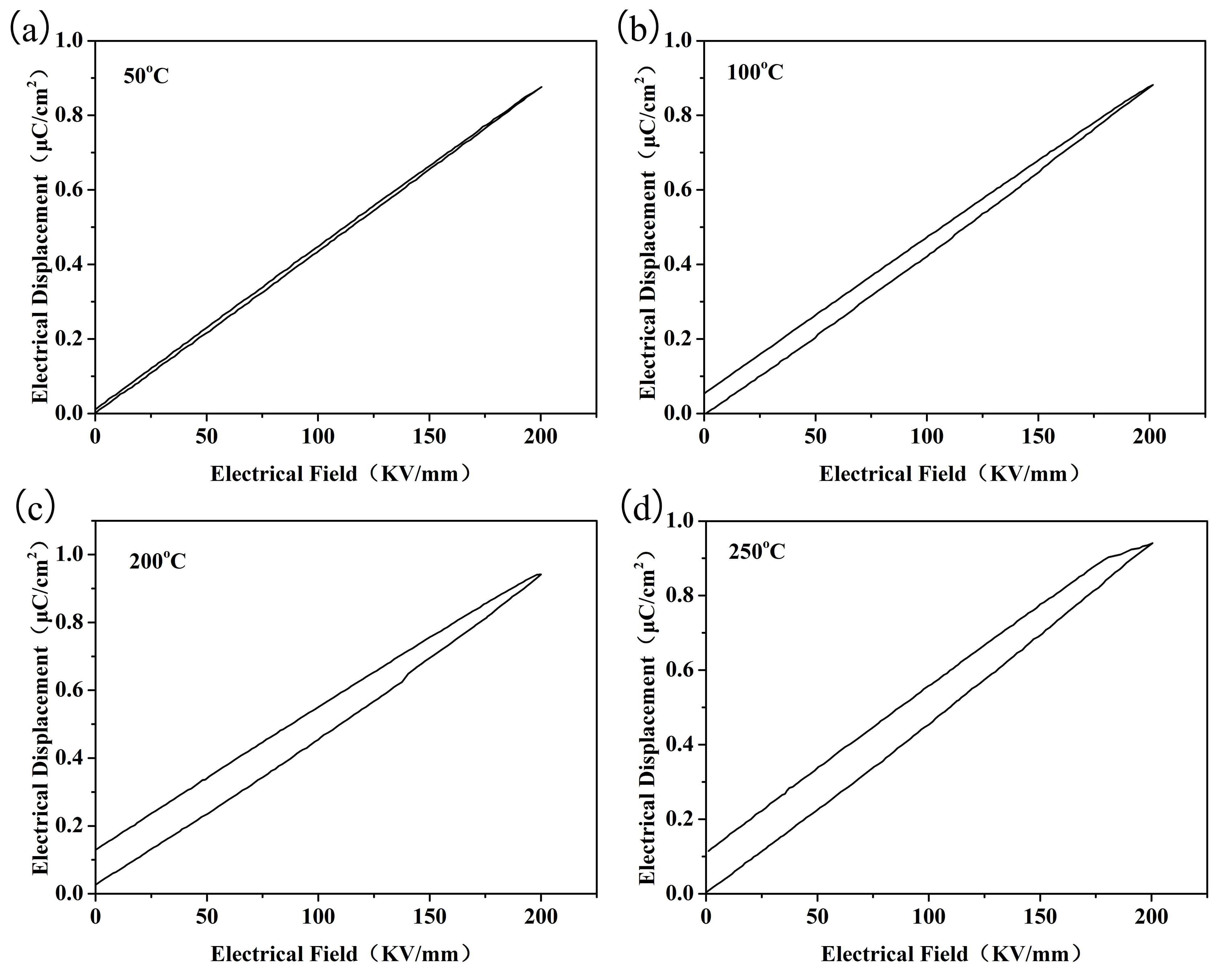


**Figure S6.** D-E loops of the PEN-Ph crosslinked films at (a) 50 ^o^C, (b) 100 ^o^C, (c) 200 ^o^C, (d) 250 ^o^C.


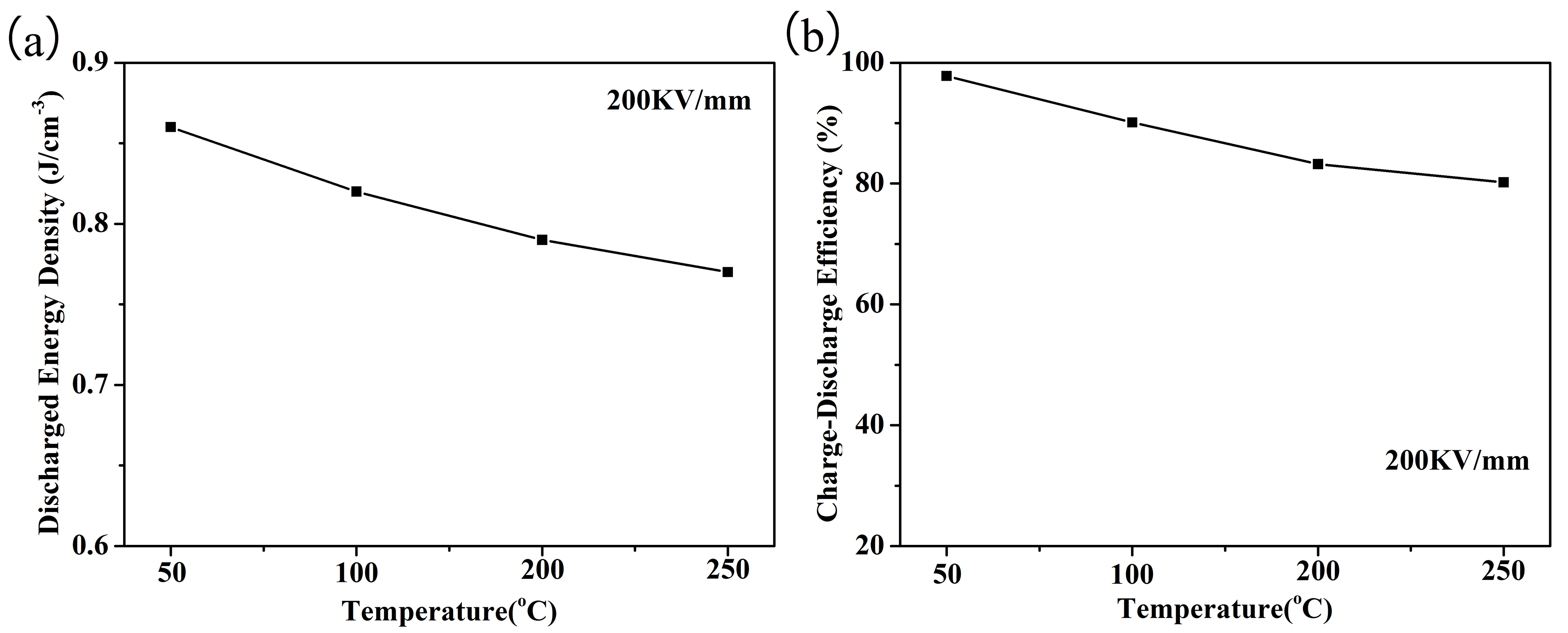


**Figure S7**. Discharge energy density as a function of temperature (a), charge-discharge efficiency of PEN-Ph crosslinked film as a function of temperature (b).

**Table S2**. Thermal and dielectric properties of BOPP and PEN-Ph self-crosslinked film.

|  | PEN-Ph | BOPP |
| --- | --- | --- |
| *T_m_* (^o^C) | Not obtain | 164.4 |
| *T_g_* (^o^C) | 385 | 86 |
| Density (g/cm^3^) | 1.25 | 0.90 |
| Modulus (MPa, 50 ^o^C) | 2200 | 1952 |
| Modulus (MPa, 150 ^o^C) | 1610 | 17 |
| Dielectric constant (1 kHz, 25 ^o^C) | 3.85 | 2.20 |
| Dielectric loss(1 kHz, 25 ^o^C) | 1.6 % | 0.12 % |
| Energy density at 200 KV/mm (J/cm^3^, RT) | 0.86 | 0.39 |
| Charge-discharge efficiency (%, RT) | 97.8 | 96.7 |
| Maximum operating temperature (^o^C) | ~300 | <150 |
